# Resistance training attenuates salt overload-induced cardiac remodeling and diastolic dysfunction in normotensive rats

**DOI:** 10.1590/1414-431X20176146

**Published:** 2017-08-07

**Authors:** D.L.M. Barretti, S.F.S. Melo, E.M. Oliveira, V.G. Barauna

**Affiliations:** 1 Laboratório de Bioquímica e Biologia Molecular do Exercício, Escola de Educação Física e Esportes, Universidade de São Paulo, São Paulo, SP, Brasil; 2 Laboratório de Fisiologia Molecular, Centro de Ciências da Saúde, Universidade Federal do Espírito Santo, Vitória, ES, Brasil

**Keywords:** Diastolic dysfunction, Interstitial collagen, Resistance training, Salt overload

## Abstract

Elevated salt intake induces changes in the extracellular matrix collagen, leading to myocardial stiffness and impaired relaxation. Resistance training (RT) has been used as a remarkably successful strategy in the treatment of heart disease. Therefore, the aim of this study was to investigate the effects of RT on preventing pathological adaptation of the left ventricle (LV) induced by salt overload. Male Wistar rats (10 weeks old) were distributed into four groups (n=8/group): control (CO), control+1% salt (CO+SALT), RT and RT+1% salt (RT+SALT). The RT protocol consisted of 4×12 bouts of squat training, 5/week for 8 weeks, with 80% of one repetition maximum (1RM). Echocardiographs were analyzed and interstitial collagen volume fraction (CVF) was determined in the LV. The 1RM tests in the RT and RT+SALT groups increased 145 and 137%, respectively, compared with the test performed before the training program. LV weight-to-body weight ratio and LV weight-to-tibia length ratio were greater in the RT and RT+SALT groups, respectively, compared with the CO group. Although there was no difference in the systolic function between groups, diastolic function decreased 25% in the CO+SALT group compared with the CO group measured by E/A wave ratio. RT partially prevented this decrease in diastolic function compared with the CO+SALT group. A 1% salt overload increased CVF more than 2.4-fold in the CO+SALT group compared with the CO group and RT prevented this increase. In conclusion, RT prevented interstitial collagen deposition in LV rats subjected to 1% NaCl and attenuated diastolic dysfunction induced by salt overload independent of alterations in blood pressure.

## Introduction

Many studies have demonstrated that salt intake is an important determinant of cardiovascular remodeling, such as ventricular hypertrophy and fibrosis (1,2). Although the effects of high salt intake were initially associated with its ability to elevate blood pressure (BP), later studies have shown that salt intake may also have cardiac effects independent of increased pressure load or sympathetic activity (3). Various mechanisms have been suggested to explain these adverse cardiovascular effects of dietary salt excess, and some evidence supports the role of the local renin-angiotensin system in their development (1).

Salt loading has been shown to induce cardiac remodeling and left ventricular (LV) dysfunction in many animal strains, at all ages, and both in normotensive and hypertensive models (4–6). Major structural alterations induced by salt loading include both LV hypertrophy and fibrosis. Of these, LV fibrosis, rather than LV hypertrophy, determinates myocardial stiffness and diastolic dysfunction (7,8).

Of the pharmacological options to prevent cardiovascular diseases, renin angiotensin system blockers and β-adrenergic receptor blockers are the most used by clinicians (9). However, the use of non-pharmacological interventions, such as exercise training, is increasing. Aerobic training (running or swimming) has been extensively prescribed to treat or prevent cardiovascular diseases, but there is as yet insufficient evidence concerning resistance training (RT) (10,11). Our group has previously characterized the cardiovascular adaptations of this type of exercise in animal models, such as concentric ventricular hypertrophy without LV internal chamber reduction and the participation of AT1R, decreased resting BP and improved single LV myocyte function (12–15). Other groups have used the same model to study the influence of RT on bone mass and insulin resistance, and as non-pharmacological therapy for the treatment of ischemia-reperfusion-induced injury (16,17).

Therefore, the aim of this study was to investigate if an RT program would prevent cardiac remodeling and LV abnormality induced by salt overload.

## Material and Methods

### Animals

Male Wistar rats (10 weeks-old) were randomly divided into four groups (n=8/group): sedentary control (CO); sedentary control plus salt diet (CO+SALT); resistance trained (RT); resistance trained plus salt diet (RT+SALT). Salt overload (1% NaCl) was administered from the adaptation period (the week before beginning the training protocol) and continued throughout the protocol in the drinking water. There were no differences in water and food intake among groups, as previously reported by our group (18). All protocols were approved by the Ethics Committee of the Escola de Educação Física e Esportes, Universidade de São Paulo, Brazil.

### Exercise protocol

**Figure 1.**
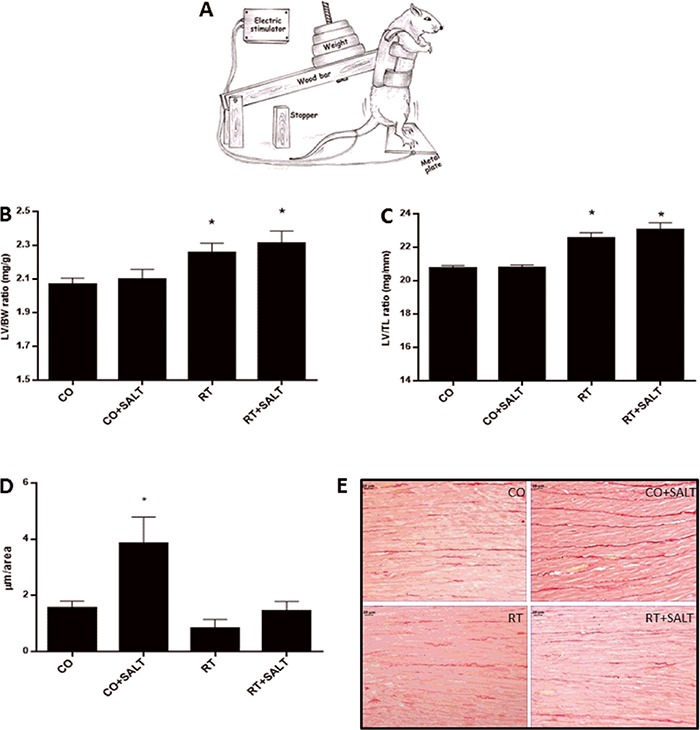
*A*, Apparatus used to perform resistance training in the rats adapted from Tamaki et al. (10); *B*, left ventricle (LV) weight-to-body weight (LV/BW) ratio (mg/g); *C*, LV weight-to-tibia length (LV/TL) ratio (mg/mm); *D*, myocardial interstitial collagen volume fraction; *E*, representative images of the collagen fibers stained with picro-sirius red in the myocardium. Data are reported as means±SE for 8 rats in each group. CO: control; CO+SALT: control+1% salt diet; RT: resistance trained; RT+SALT: resistance trained+1% salt diet. *P<0.05 *vs* CO and CO+SALT. For all variables, one-way ANOVA followed by Duncan *post hoc* test was used.

Animals were exercised following a model adapted from Tamaki et al. (19) (Figure 1A) and previously used by our group (12). The rats wore canvas jackets to enable regulation of the twisting and flexion of their torsos, and they were suspended in a standard position on their hind limbs. Electrical stimulation (20 V, 0.3-s duration, at 3-s intervals) was applied to the tail through a surface electrode. Stimulated rats flexed their legs repeatedly, which lifted the weight arm of the training apparatus. The rats were trained by 4×12 repetitions, with a 90-s rest period between each set for 8 weeks. The animals previously underwent adaptation for 1 week (1× day, with a load of 200 g) and on the last day of adaptation and every two weeks, the maximum weight lifted [one repetition maximum (1RM)] was measured and the training load was set at 80% of this value (19). The 1RM was defined as the minimum load at which the rats were unable to jump following electrical stimulation. Exercise training sessions were performed in the morning (8:00–10:00 am) in a dark room. Our previous study demonstrated that this electrical stimulation does not change catecholamines, plasma levels, or adrenal weight as stress markers (14).

### Echocardiography

Echocardiography analysis was performed in accordance with the recommendations of the American Society of Echocardiography and following previous publications by our group (15). Echocardiography was performed 24 h after the last training session and before surgery to introduce the catheter into the carotid artery. Transthoracic echocardiography was performed after the experimental period, and was based on the average of three consecutive cardiac cycles using Sequoia 512 equipment (ACUSON Corporation, USA) with a 10- to 14-MHz multifrequency linear transducer placed on the animal's shaved chest (lateral recumbence). Rats were anesthetized with ketamine (90 mg/kg) and xylazine (10 mg/kg). Two-dimensional parasternal long-axis and short-axis views were recorded, in addition to two-dimensional targeted M-mode traces through the anterior and posterior LV walls. LV end-diastolic internal diameter (LVIDd), diastolic posterior wall thickness (PWTd), diastolic interventricular septum thickness (IVSTd), and LV mass (LVM) were determined. LV diastolic and systolic diameters were measured from M mode or 2-dimensional images of the mid-LV chamber. In addition, both diastolic and systolic transverse areas of the LV were measured by 2-dimensional echocardiography at basal, mid-view, and apical views. The final value was the arithmetic mean of the measures of the three views. Systolic function was analyzed by the LV fractional shortening (FS), LV ejection fraction (EF), and velocity of circumferential fiber shortening (VCF). Diastolic function was evaluated based on the parameters of mitral inflow and LV outflow tract velocity curves, using pulsed wave Doppler and with tissue Doppler imaging of the mitral lateral annulus. From the mitral diastolic flow velocity curve, the maximum velocity of the early (E) and late (A) waves were measured and the E/A ratio calculated. Tissue Doppler imaging was performed to measure the E and A diastolic wave velocities of the lateral mitral annulus from a 4-chamber apical view.

### Arterial BP and heart rate (HR)

Twenty-four hours after the last training session and under pentobarbital anesthesia (40 mg/kg, *ip*), a catheter (PE-50) was inserted into the carotid artery, emerging through the back of the rat's neck. During the experimental session, this catheter was connected to a strain-gauge transducer (P23 Db; Gould-Statham Instruments Inc., USA). Twenty-four hours after the surgery, arterial BP was recorded on a beat-to-beat basis (AT/CODAS) at a frequency of 100 Hz for 30 min in quiet, conscious, unrestrained rats. The data reported indicate the average of all values of systolic, diastolic, and mean arterial pressure over the entire recording period of 30 min. HR was taken from the BP pulse.

### Ventricular hypertrophy

Ventricular hypertrophy was determined by the ratio of LV weight to animal body weight and the ratio of LV weight to tibia length.

### Interstitial collagen volume fraction

The LV was fixed in 6% formaldehyde and embedded in paraffin, then cut into 5-µm sections at the level of the papillary muscle. The LV was stained with picro-sirius red and fields were selected from sections placed in a projection microscope, as reported previously (11). The collagen volumetric fraction (CVF) was calculated as the sum of all connective tissue areas divided by the sum of all muscle areas in all fields. All measurements were performed by the same observer who was blind to group allocation.

### Statistical analysis

Statistical analysis was performed using one-way analysis of variance (SigmaStat 4.0 software, USA) except for body weight and 1RM which were compared using two-way analysis of variance. Duncan's *post hoc* test was used for individual comparisons between means when a significant change was observed. A level of significance of P<0.05 was adopted for all experiments. Data are reported as means±SE.

## Results

### Body weight (BW)

In the CO and CO+SALT groups, BW increased by 15.6 and 14.5%, respectively (P<0.05). No statistically significant increase was observed in either the RT (9.6%) or RT+SALT (8.0%) groups (Table 1).

**Table 1.** Body mass, absolute 1RM, systolic and diastolic blood pressure, and heart rate.

Data	CO	CO+SALT	RT	RT+SALT
Body weight (g)				
1st week	396±8	370±7	383±5	386.8±6
8th week	458±3*	424±5*	420±8	417.8±0
1RM (g)				
1st week	889±22	910±5	924±19	1011±44
8th week	1010±84	1080±72	2271±74*	2400±105*
Systolic blood pressure (mmHg)	123±3	126±4	122±4	119±9
Diastolic blood pressure (mmHg)	101±4	99±3	98±5	97±8
Heart rate (bpm)	341±8	344±10	325±11	326±9

Data are reported as means±SE for 8 rats in each group. Body weight and 1RM values were measured in the 1st and 8th weeks of the training protocol. Direct measurement of the systolic and diastolic blood pressure and heart rate were performed 48 h after the last training session. 1RM: 1 repetition maximum; CO: control; CO+SALT: control+1% salt diet; RT: resistance-trained; RT+SALT: resistance-trained+1% salt diet.

* P<0.05, 8th *vs* 1st week. For body weight and 1RM, two-way ANOVA followed by Duncan *post hoc* test was used. For blood pressure and heart rate, one-way ANOVA followed by Duncan *post hoc* test was used.

### Maximal strength

In the initial 1RM test, all groups lifted a similar weight (Table 1). Both the RT (145%) and RT+SALT (137%) groups increased their maximal 1RM at the end of the protocol (Table 1). No significant changes were observed in the sedentary groups (CO, 13.6%; CO+SALT, 18.6%). The absolute 1RM-to-body weight ratio shows that the animals began lifting ∼2-fold of their BW and finished the training period lifting ∼4-fold of their BW (Table 1).

### BP and HR

Systolic BP, diastolic BP, and HR remained similar among groups, regardless of treatment (Table 1).

### Ventricular hypertrophy

The LV weight-to-body weight ratio was 8.5 and 10.6% greater in the RT and RT+SALT groups, respectively, compared to the CO group (Figure 1B). Similarly, the LV weight-to-tibia length ratio was 8.6% and 10.5% higher in the RT and RT+SALT groups, respectively, than the CO group (Figure 1C). Left ventricular mass (Table 2), measured by echocardiography, was 12 and 19% higher in the RT and RT+SALT groups, respectively, than the CO group. There was no difference between the CO and CO+SALT groups or the RT and RT+SALT groups.

**Table 2.** Heart rate, cardiac structure, systolic and diastolic left ventricle (LV) function by echocardiography.

Data	CO	CO+SALT	RT	RT+SALT
Heart rate (bpm)	321±4	310±16	318±9	312±12
LVIDd	0.74±0.04	0.75±0.08	0.76±0.06	0.77±0.07
PWTd	0.144±0.006	0.140±0.004	0.155±0.005#	0.158±0.004#
IVSTd	0.143±0.007	0.141±0.005	0.154±0.003#	0.157±0.004#
LVM (g)	0.68±0.04	0.67±0.05	0.76±0.03#	0.83±0.04#
Systolic function				
FS (%)	35.2±0.8	33.4±1.1	36.8±1	36.2±1.4
EF (%)	71.8±1.4	67.8±1.5	72.2±1.2	72.4±1.8
VCF (m/s)	4.5±0.1	4.3±0.2	4.5±0.2	4.8±0.2
Diastolic function				
E-wave (m/s)	0.571±0.012	0.572±0.023	0.570±0.017	0.553±0.013
A-wave (m/s)	0.315±0.008	0.419±0.015*	0.306±0.008	0.360±0.014
E/A ratio	1.81±0.01	1.37±0.03*	1.87±0.05	1.55±0.05* +

Data are reported as mean±SE for 8 rats in each group. Echocardiography was performed 24 h after the last training session. CO: control; CO+SALT: control+1% salt diet; RT: resistance-trained; RT+SALT: resistance-trained+1% salt diet; LVIDd: left ventricular end-diastolic internal diameter; PWTd: diastolic posterior wall thickness; IVSTd: diastolic interventricular septum thickness; LVM: left ventricular mass; FS: left ventricular fractional shortening; EF: left ventricular ejection fraction; VCF: velocity of circumferential fiber shortening; E and A: early and late waves.

* P<0.05 *vs* CO and RT;

+ P<0.05 *vs* CO+SALT;

# P<0.05 *vs* CO and CO+SALT (one-way ANOVA followed by Duncan *post hoc* test).

### Ventricular function

The echocardiographic results are summarized in Table 2. There was no systolic dysfunction among the groups in terms of shortening fraction, ejection fraction, and velocity of circumferential fiber shortening. In contrast, we observed diastolic dysfunction markers. Although there was no change in the peak E-wave velocity, the CO+SALT group showed increased peak A-wave velocity, which resulted in a 25% decrease in the E/A ratio compared to the CO group, while RT attenuated diastolic function by 12% in the RT+SALT group compared with the CO+SALT group (Table 2). E/A represents the ratio of early wave to late wave ventricular filling velocities. In the healthy heart, E velocity is greater than the A velocity (20). However, in the condition of LV wall stiffness there is a change in these filling velocities, lowering the E/A ratio. A velocity being greater than E velocity is often accepted as a marker of diastolic abnormality due to impaired LV filling (20).

### Interstitial CVF

The salt diet induced more than a 2.4-fold increase in CVF in the CO+SALT group compared with the CO group (Figure 1D). Interestingly, RT abrogated this increase in the RT+SALT group, maintaining a CVF similar to that in the CO group. Figure 1E shows representative images of the collagen fibers stained with picro-sirius red in the LV.

## Discussion

This study demonstrated that RT attenuated LV diastolic dysfunction induced by adding 1% salt in the diet. The main findings of the study are that RT prevented LV interstitial collagen formation in rats subjected to 1% NaCl and attenuated diastolic dysfunction development independent of alterations in BP.

There are many studies in the literature reporting morphological LV changes induced by a salt diet independent of increased BP (2). As expected, our 1% NaCl in drinking water did not affect BP (18). Furthermore, we did not observe a change in LV mass induced by the 1% salt diet (CO+SALT group). Although the CO+SAL group did not develop either ventricular hypertrophy or hypertension, we observed LV dysfunction and increased interstitial collagen fraction in the myocardium. It has been shown that a high-salt diet in normal mice induces activation of the local cardiac renin-angiotensin system, which is the main factor in the myocardial production of pro-fibrotic factors such as aldosterone and angiotensin II (1). In line with the findings described above, Baldo et al. (21) showed that spontaneously hypertensive rats treated with a high-salt diet develop LV stiffness, which is prevented by the aldosterone receptor blocker spironolactone, suggesting that local aldosterone produced in the myocardium may mediate salt-induced fibrosis. Furthermore, other factors, such as transforming growth factor-b1 (TGF-b1), have been shown to induce myocardial fibrosis in normotensive rats treated with salt (22).

A previous study by Doustar et al. (17), using another RT model (climbing a vertical ladder), showed that 4 weeks of RT did not preserve the heart against ischemia-reperfusion injury, evidenced by no change in the infarct size. Quinteiro et al. (23) showed that only eight weeks of dynamic aerobic exercise training, but not RT (climbing a vertical ladder), was able to attenuate systolic and diastolic dysfunction in postmenopausal rats with diabetes. Similarly, RT (in-water jump) for five weeks was not effective in reversing fetal gene re-expression or LV remodeling induced by supra-physiological doses of anabolic steroids (24). On the other hand, Soufi et al. (16) showed that 12 weeks of RT (squat training model) provides cardioprotection in a coronary artery disease model. In addition, Alves et al. (25) also showed that 12 weeks of RT (squat training model) in a chronic heart failure model was able to improve cardiac function, and attenuate ventricular hypertrophy and LV CVF. Furthermore, Mostarda et al. (26) showed that 10 weeks of low-intensity RT (squat training model) increased systolic function in diabetic rats due to positive LV remodeling and improved baroreflex sensitivity. In summary, positive effects on LV function were observed in those studies in which the RT protocol lasted longer than eight weeks and those that used the squat-training apparatus, similar to that used here. We may speculate that the training workload, the muscular mass used, or the type of muscular contraction involved in each RT model could explain the divergent results in each model.

To the best of our knowledge, there are no studies exploring the mechanism between collagen remodeling and diastolic function after RT training. However, our group showed the expression of microRNA-29c, which targets the collagen gene and is associated with less collagen deposition in heart tissue in swimming exercise. In addition, microRNA-29a and -29c expression prevented collagen type I and III expression in the border and the remote regions of the myocardial infarction. In these two studies (11,27) there was an improvement in the diastolic function, as shown by the E/A ratio obtained by echocardiography.

Although experimental studies have focused on the pathogenic effects of salt via its ability to elevate BP, it has recently become evident that salt appears to play an important and direct role in myocardial structure and function. We believe that RT may prevent the deleterious effects of salt overload by decreasing collagen deposition and thus avoiding LV stiffness. The findings shown here in rodents should be reproduced in humans and thus RT may become an important non-pharmacological strategy in the treatment of heart disease, similar to aerobic training.

As study limitations, we should point out that water intake was not measured, which does not allow us to know the exact amount of salt intake. Also, interstitial fibrosis was only measured by microscopy and future studies should dissect the molecular mechanisms behind RT modulation of salt effects independent of BP changes.

In conclusion, we show for the first time that RT prevented interstitial collagen deposition in LV rats subjected to 1% NaCl and attenuated diastolic dysfunction induced by salt overload independent of alterations in BP.
